# The EurOPDX Data Portal: an open platform for patient-derived cancer xenograft data sharing and visualization

**DOI:** 10.1186/s12864-022-08367-1

**Published:** 2022-02-22

**Authors:** Zdenka Dudová, Nathalie Conte, Jeremy Mason, Dalibor Stuchlík, Radim Peša, Csaba Halmagyi, Zinaida Perova, Abayomi Mosaku, Ross Thorne, Alex Follette, Ľuboslav Pivarč, Radim Šašinka, Muhammad Usman, Steven Neuhauser, Dale A. Begley, Debra M. Krupke, Massimiliano Frassà, Alessandro Fiori, Riccardo Corsi, Luca Vezzadini, Claudio Isella, Andrea Bertotti, Carol Bult, Helen Parkinson, Enzo Medico, Terrence Meehan, Aleš Křenek

**Affiliations:** 1 Institute of Computer Science, Masaryk University, Šumavská 15, 60200 Brno, Czech Republic; 2 European Molecular Biology Laboratory- European Bioinformatics Institute, Wellcome Trust Genome Campus, Hinxton, Cambridge, CB10 1SD UK; 3 The Jackson Laboratory, 600 Main Street, Bar Harbor, ME 04609 USA; 4 Department of Oncology, University of Torino, 10060 Candiolo, TO Italy; 5 Kairos3D, via Agostino da Montefeltro 2, 10134 Turin, Italy; 6 Candiolo Cancer Institute, FPO-IRCCS, S.P. 142, km 3,95, 10060 Candiolo, TO Italy

**Keywords:** PDX, Molecular data analysis, Data harmonization, Research infrastructure, Database

## Abstract

**Background:**

Patient-derived xenografts (PDX) mice models play an important role in preclinical trials and personalized medicine. Sharing data on the models is highly valuable for numerous reasons – ethical, economical, research cross validation etc. The EurOPDX Consortium was established 8 years ago to share such information and avoid duplicating efforts in developing new PDX mice models and unify approaches to support preclinical research. **EurOPDX Data Portal** is the unified data sharing platform adopted by the Consortium.

**Main body:**

In this paper we describe the main features of the EurOPDX Data Portal (https://dataportal.europdx.eu/), its architecture and possible utilization by researchers who look for PDX mice models for their research. The Portal offers a catalogue of European models accessible on a cooperative basis. The models are searchable by metadata, and a detailed view provides molecular profiles (gene expression, mutation, copy number alteration) and treatment studies. The Portal displays the data in multiple tools (PDX Finder, cBioPortal, and GenomeCruzer in future), which are populated from a common database displaying strictly mutually consistent views.

**(Short) Conclusion:**

EurOPDX Data Portal is an entry point to the EurOPDX Research Infrastructure offering PDX mice models for collaborative research, (meta)data describing their features and deep molecular data analysis according to users’ interests.

## Background

Patient-derived xenografts (PDX) become increasingly important as they provide a new approach for preclinical cancer research. PDX are in vivo models in which human cancer tissues are implanted in animal hosts, typically immunocompromised mice [1]. Exploration of cancer molecular features and drug response in PDX models has yielded a huge amount of information, in some cases leading to changes in cancer patient management [2, 3].

Since the number of researchers working with PDX mice models across Europe is rising quickly, as well as the number of models generated, there was a need to organize information about European PDX mice models. To avoid duplication of efforts, enable extensive collaboration, and to set up shared standards suitable for the PDX mice model facilities in Europe and beyond (to allow for cross-validation studies, among other reasons), the EurOPDX Consortium was established in 2013. Sharing biospecimens, data sets and standardization of laboratory procedures following high quality standards have been the main objectives of the Consortium [2].

To fulfill these goals, EurOPDX has started building a distributed infrastructure for PDX research providing services to other research groups, including PDX mice models biobanking and delivery, as well as drug efficacy testing. The first step towards construction of the EurOPDX Research Infrastructure (RI) has been the joint collection, harmonization and display of PDX-related data from partners, according to existing and newly developed standards. The work we present here is the current status of the **EurOPDX Data Portal**^1^ which enables integrative search, browsing and exploration of more than a thousand PDX models for preclinical, clinical and molecular information.

## Construction and content

### Main features of the EurOPDX Data Portal

Through its Data Portal, the EurOPDX Consortium provides access to three main types of PDX-related data: (i) metadata, i.e., all annotations related to PDX model construction, tumor of origin, preclinical and clinical features as specified in the PDX-Minimal Information (PDX-MI) standard [4], (ii) processed molecular data, including cytogenetics and gene expression, mutation and copy number alterations, (iii) additional data, such as model’s drug dosing and patient treatment data.

**Fig. 1:**
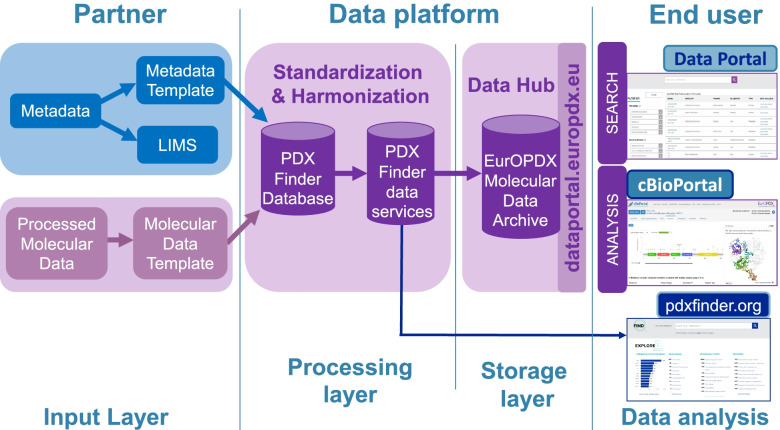
EurOPDX Data Portal data flows. Four layers are described: (i) Input layer for metadata and processed molecular data provided by partners from the consortium. These data are collected via templates and laboratory information management systems (LIMS); (ii) Data processing layer where the data are collected, semi-automatically standardized and harmonized to be aligned with PDX Finder data services utilized in the Portal as well; (iii) Storage layer consists of the Data Hub where the EurOPDX data are stored; (iv) Data analysis layer is then a graphical user interface enabling (meta)data browsing and further analysis

Figure 1 illustrates the data flow, from partners owning and providing the models and related data, to the end user of the Portal, through the Data Platform.

The current data loading process is semi-automatic and occurs through four main steps: (i) EurOPDX data providers fill in a metadata template. (ii) The data is checked for consistency and harmonized (e.g., unifying the gene symbols, diagnoses identifiers etc.) before ingesting into the database. Any additional datasets such as processed molecular data or drug dosing studies are collected in separate files. (iii) Metadata template and datasets are processed and validated by loader components developed within the PDX Finder catalogue [5], (iv) and uploaded into the EurOPDX Data Hub. Details on the whole process are given in the following sections. Technically, the same process and software is used to populate the database of the standalone PDX Finder^2^ (see Fig. 1).

Currently, the Portal displays models and data for a total of **1010 PDXs from 9 PDX providers**, across seven European Countries (Table 1).

**Table 1:** Overview of EurOPDX partners providing the data sets, cancer types and number of PDX mice models represented by these data sets

Institute (acronym)	Cancer Type (data set acronym)	Number of PDX Models
Candiolo Cancer Institute, FPO-IRCCS (IRCC), Italy	Colorectal Cancer (IRCC-CRC)	639
Candiolo Cancer Institute, FPO-IRCCS (IRCC), Italy	Gastric Cancer (IRCC-GC)	76
Cancer Research UK, University of Cambridge, UK	Breast Cancer (UOC-BC)	59
Curie Institute - Preclinical Investigation Laboratory, France	Breast Cancer (Curie-BC)	5
Curie Institute - Preclinical Investigation Laboratory, France	Lung Cancer (Curie-LC)	6
Curie Institute - Preclinical Investigation Laboratory, France	Ovarian Cancer (Curie-OV)	5
Luxembourg Institute of Health - NORLUX laboratory	Glioma (LIH)	40
Netherlands Cancer Institute	Breast Cancer (NKI)	7
TRACE - Patient Derived Tumor Xenograft Platform, KU Leuven and UZ Leuven, Belgium	Breast Cancer, Cutaneous Melanoma, Uterine Cancer (TRACE)	31
Vall d’Hebron Institute of Oncology, Spain	Breast Cancer (VHIO-BC)	5
Vall d’Hebron Institute of Oncology, Spain	Colorectal Cancer (VHIO-CRC)	74
Vall d’Hebron Institute of Oncology, Spain	Pancreatic Cancer (VHIO-PC)	43
University of Manchester, UK	Breast Cancer (UOM-BC)	12
University Medical Center Groningen, Netherlands	Ovarian Cancer (UMCG)	8

These models represent several cancer systems listed in Table 2.

**Table 2:** PDX models available at the EurOPDX Data Portal listed according to cancer system

Cancer System	Number of PDX Models
Digestive system	842
Breast	346
Nervous system	40
Reproductive system	21
Connective and soft tissue	8
Skin	8
Thoracic	6

Most of the models represent adult cancer models (93%), in 7% of cases age was not specified by the provider.

**Fig. 2:**
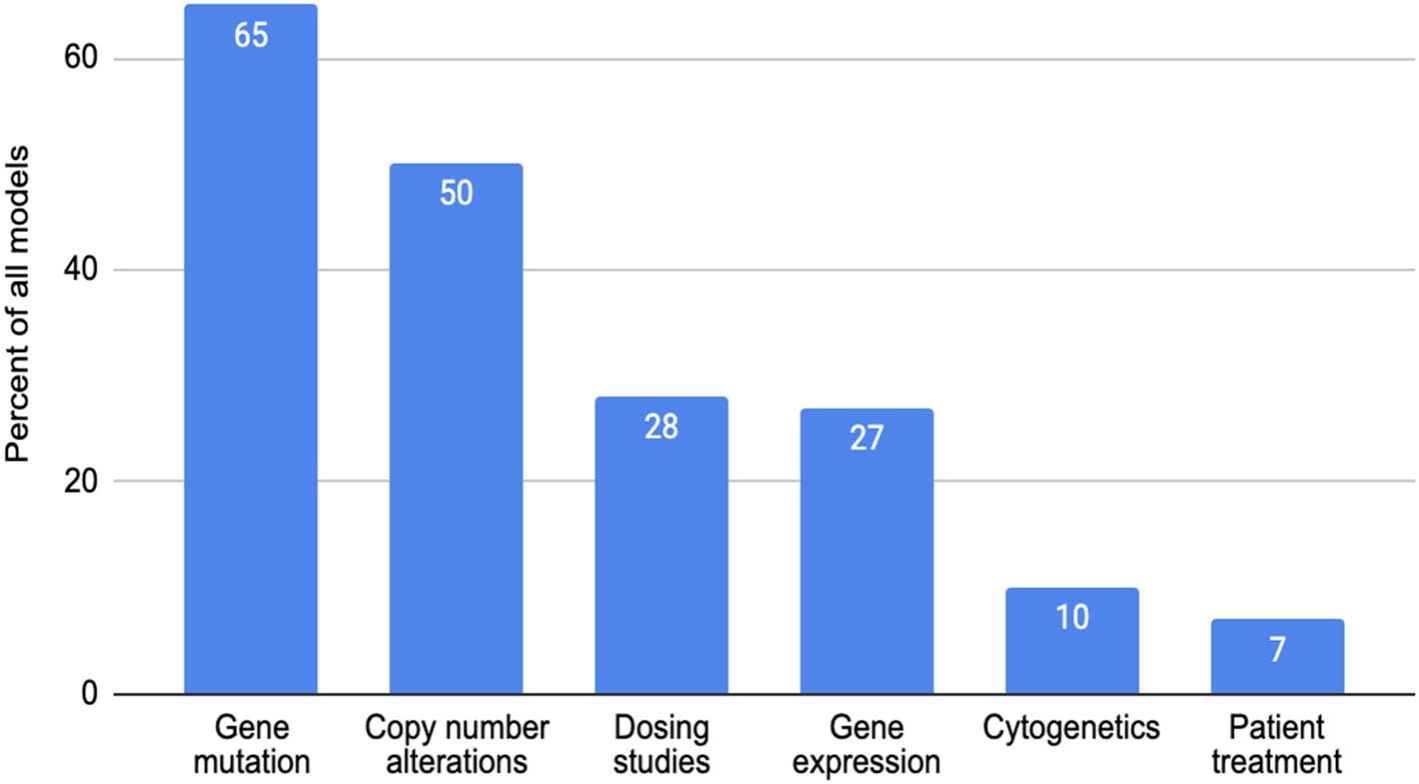
Visualization of data types available in the Data Portal, by percentage. Out of 1010 deposited models, 653 include gene mutation data (65%), 505 include copy number alteration data (50%), for 283 models there are available drug dosing studies (28%), 270 models include gene expression data (27%), 103 models cytogenetics (10%), and 69 models include patient treatment data (7%)

Figure 2 shows coverage of the models by additional data (molecular, treatment etc.). Gene mutation and copy number alteration are already available for half of the models or more, while other data are being introduced gradually.

EurOPDX Data can be browsed and retrieved by the end user through two main graphical interfaces: the Data Portal Search Interface^3^ and the EurOPDX cBioPortal Interface.^4^ While the Search interface allows mainly searching models by metadata, the cBioPortal interface allows in-depth search and analysis of gene-level molecular data [6, 7], see examples at Fig. 5.

### Input layer

Metadata is collected via Excel templates as this format has been the most preferred based on user feedback. The European Molecular Biology Laboratory - European Bioinformatics Institute (EMBL-EBI) and University of Turin teams developed and refined 9 collection templates to ensure a global community coverage and compliance with the PDX-MI standard [4]. This standard defines the minimal information for describing the clinical attributes of a patient’s tumor, the details of model creation, quality assurance methods, and metadata associated with the model’s availability for use in cancer research. These templates have been re-used by the global PDX community and new templates are developed or updated on an ad hoc basis to cover new data types or attributes. Current templates include:

PDX mice models and sample Metadata templates

- Basic Metadata template collecting patients’ clinical and PDX mice models metadata
- Sample Metadata template collecting metadata for molecular data

Individual templates for data (molecular and treatment)

- Molecular datasets templates
  - ° Cytogenetics
  - ° Mutation
  - ° Copy number alterations
  - ° Fusion
  - ° Transcriptomic
- Treatment template (patient and PDX mice models)
  - ° Patient treatment
  - ° Drug Dosing of the PDX mice models

### Processing layer

Metadata provided by partners are validated for adherence to the PDX-MI standard [4]. Metadata harmonization is achieved by mapping biologically identical histological concepts provided by different sources. Specifically, to support consistent searching across resources, we use different attributes such as original histological term and the primary tissue provided by the resource. For example, histological concepts “Adenosquamous”, “adenosquamous carcinoma”, “Ad and SC carcinoma” share the same primary tissue “lung” and are mapped to the National Cancer Institute thesaurus (NCIt) [8] ontological label “Adenosquamous Lung Carcinoma”. Moreover, concepts are aggregated based on meaningful groupings like cancer by anatomical system or cell morphology. This approach allows a search for “lung cancer” models to display hits across all subclasses of lung cancer models in a single query.

Processed Molecular Data Sets are retrieved by the EMBL-EBI team from existing repositories/locations as provided by the data owners. Data is then validated and uploaded to a Neo4J database (details in the following section). To populate the database a bespoke Java Extraction-Transformation-Loading (ETL) pipeline was written to extract relevant attributes corresponding to the PDX-MI standard from the data provided by the PDX mice models providers. Software is freely available on GitHub^5^ under an Apache 2.0 license.^6^

A major bottleneck to molecular data integration is the heterogeneity of the data produced from multiple sources that involves a variety of sequencing platforms, laboratory protocols and analyses. This heterogeneity introduces strong technological biases and causes data inconsistencies, hindering data integration efforts. To control this technical variability, we have developed a service to remap all PDX mutation data to the same genome assembly (GRCh38). We then re-annotated variants on a single annotator (Variant Effect Predictor – VEP^7^). This harmonization provides accurate and standardized annotation of variants, following Human Genome Variation Society (HGVS) standards,^8^ and it ensures consistent searching and linking to cancer annotation databases like CivicDB [9], OpenCravat [10], and COSMIC [11].

In addition to the PDX-MI standard, data from the models in the EurOPDX Data Portal comply with other nomenclature and metadata standards accepted by the community: NCI thesaurus [8] for cancer type, diagnosis and other cancer attributes, Human Genome Organization Gene Nomenclature Committee [12] standards for the names and symbols of the human genes, and International Committee on Standardized Genetic Nomenclature for Mice [13] for host mouse strain nomenclature. NCIt [8], ChEBI [14], CHEMBL [15] and PubChem [16] are used to standardize drugs and compound names.

### Storage layer

**Fig. 3:**
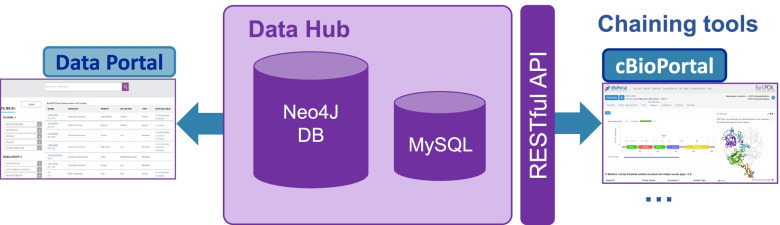
EurOPDX Data Hub schema. The Hub includes the Neo4J database containing data accessible via the Data Portal Search page, and the MySQL database providing temporary operational data. There is a REST API available within the Hub accessible to the chaining tools

The EurOPDX Data Hub constitutes the repository layer of the RI and provides an application programming interface (API) for exporting selected data sets to chaining tools like cBioPortal, see Fig. 3.

The Data Hub is designed to handle multiple types of datasets and databases. Three types of data are stored:

1. **Clinical data** that contain pseudonymized information about patients (e.g., age at the collection, sex, diagnosis) and about PDX mice models (stage of cancer, site of the tumor, primary/metastasis status).
2. **Metadata** which describe how and where the PDX mice models were created/prepared (e.g., implant site of engraftment, an identifier of the mouse).
3. **Genomic data** – this comprises an enormous set of different data types. Currently expression, copy number alteration, and mutation.

**Fig. 4:**
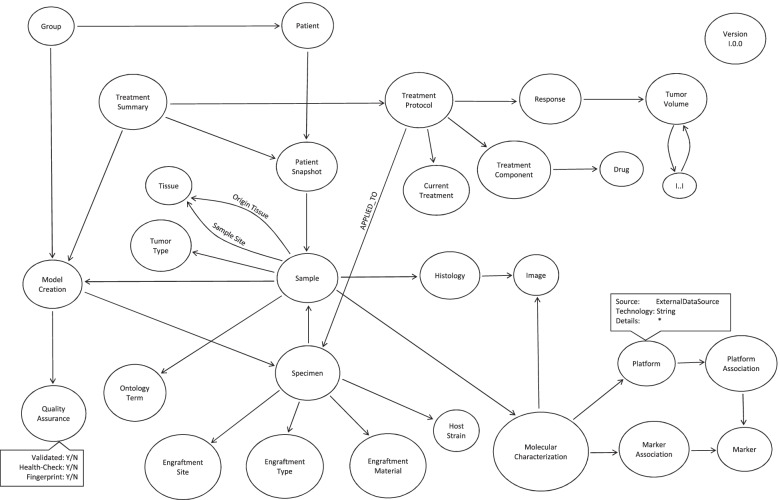
Schema of the Neo4J Database. Each node of the graph database schema is an entity or event captured in the data. Arrows, so called edges, show the relationships between the nodes

The storage is based on a Neo4J database (DB) that follows the structure from PDX Finder’s [5] Neo4J DB records of EurOPDX partners/data providers (the N4J DB schema is shown in Fig. 4) models, patients, clinical data, and molecular data. Besides Neo4J there is a MySQL DB for storing temporary data in Data Hub, and eventual extensions not covered by the PDX Finder data schema (it is not required currently but it was the case of the previous version before gene expression data became supported by PDX Finder).

Data loading tools of PDX Finder – including automatized standardization and harmonization – are used for loading data to the shared database of the Hub. The Hub design is not restricted to using the Neo4J database only. On the contrary, the previous versions complemented it with a relational database to store additional data, being populated with other tools. The current setup is the result of unification developments.

The RESTful API^9^ of the Hub provides a unified way for export of clinical data, metadata and molecular data for a specified set of PDX models. This API is designed to be complete in the sense that any chained visualization on data processing tool (cBioPortal currently, GenomeCruzer^10^ foreseen) can retrieve *all data it needs* via this API. In this way, strict consistency of the data among the tools is ensured. Technically, we manage the tool integration by wrapper scripts, which retrieve the data using the API, format them appropriately, and feed to the wrapped tools.

The Data Hub API is based on Python’s Flask framework. To ensure automated deployment and effective maintenance, the Data Hub runs with its components (Neo4J, MySQL, API and Proxy) within Docker containers managed by docker-compose [17].

### Output layer

The user interface of the above-described database is integrated with EurOPDX Data Portal web pages in Umbraco Content Management System [18]. The principal entry point^11^ is based on branded PDX Finder software with minor extensions (classifying models by their availability for Trans-national access via the EurOPDX RI, search by specific model identifiers, etc.). This interface allows search and selection of the models by metadata (origin, diagnosis, treatment, etc.). An example of the search screen is shown in Fig. 6.

**Fig. 5:**
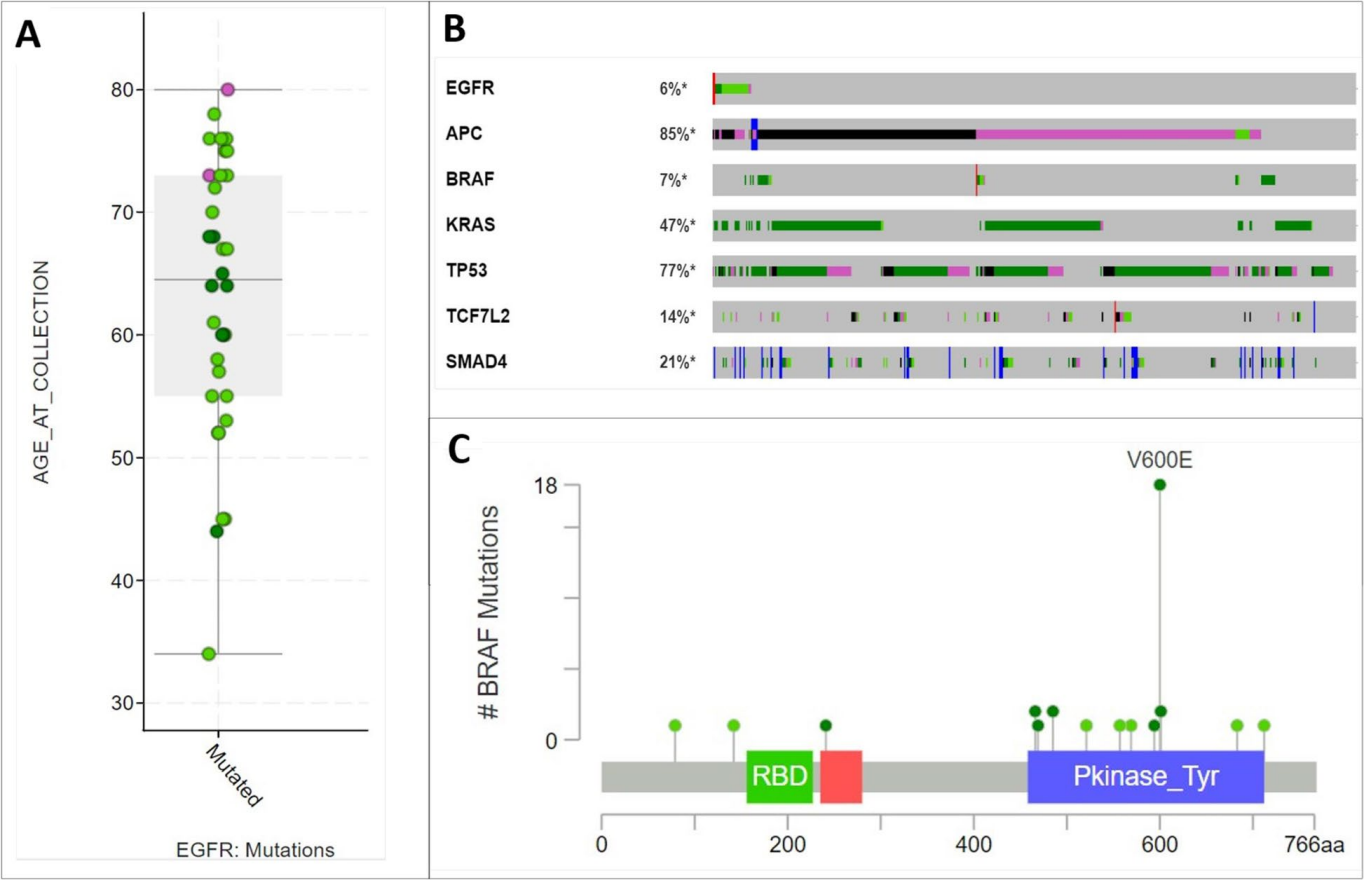
EurOPDX data visualization in the EurOPDX cBioPortal instance. **A** Example of plots which can be designed according to the user’s needs. This is a plot showing EGFR gene mutations compared with age of the patient within a selected study; **B** OncoPrint of queried genes. Provides information about number of samples per patient within a selected study, data types availability per each sample and gene mutations or alterations; **C** Example of mutation analysis of a chosen dataset – MutationMapper tool. It is possible to browse mutations within the selected gene - mutations mapping on a linear protein and its domains, type of the mutation – and get detailed sample information in which mutations are present. 3D visualizations of the protein interactions and other mutation information is available by linking external databases

The same data are available by the EurOPDX cBioPortal interface, populated by a setup procedure which calls the Data Hub API as described above. This interface is focused on more complex analyses and visualizations of the molecular data (gene expression charts, mutations, and their correlations), see Fig. 5 for several examples of the visualization tools available within the cBioPortal platform.

### User registration and authentication

The Portal is freely accessible to any researcher from the internet after a lightweight, self-service registration, required for maintaining minimalistic records of the service usage enforced by policies of the underlying cloud service provider, as well as to keep track of users’ agreement to the terms of Acceptable Use Policy (which is, besides forbidding unlawful use, mostly informative only, not restricting the use of data for non-commercial purposes), and to offer personalized services eventually. We adopted the technical solution developed in the ELIXIR research infrastructure [19], which merges the possibility to use the user identity of any institution supporting the eduGAIN^12^ federation, as well as social identities (Google, LinkedIn, ORCID); migration to the emerging European Life-Science authentication infrastructure is foreseen.

## Utility and discussion

### User interface

Users can reach the EurOPDX Data Portal homepage at https://dataportal.europdx.eu/. This page displays information about competitive calls by the EurOPDX RI for access to PDX models, model data availability and a link to the Search page at https://dataportal.europdx.eu/search.

User registration/login is required to access the Search page.

**Fig. 6:**
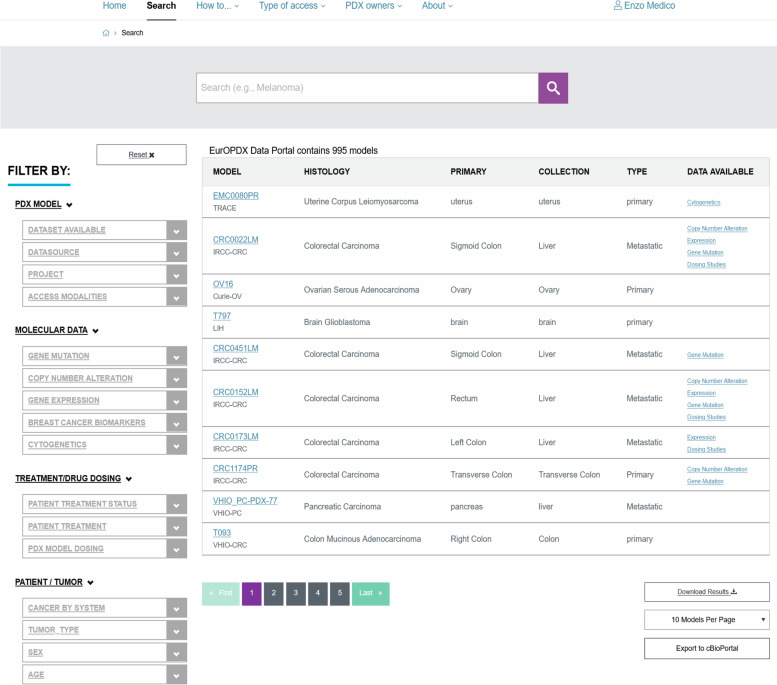
Screenshot of the Search page from the EurOPDX Data Portal

As Shown in Fig. 6, the Search page is subdivided into two sections. On the left, multiple filters allow selection of models by basic features, molecular data, treatment data, and patient/tumor data. Each filter category is further subdivided into sub-categories, and the filters can be mixed for accurate queries based on the user’s needs. For users interested in accessing models through the EurOPDX RI, a filter enables selection of models available for Trans-national access.^13^ Models are also accessible on a collaborative basis, by contacting the PDX owners. The right part of the Search page displays the results of the query in tabular format, with each model in a separate row. Key features of the models are presented in columns and include model identifier (ID) and its original provider, tumor histological classification, site of primary tumor, collection site, and links to available datasets (Table 3).

**Table 3:** Links to publicly available datasets

Institute (acronym)	Cancer Type (data set acronym)	Relevant PDX Models	Link to data
Curie Institute - Preclinical Investigation Laboratory, France	Ovarian Cancer (Curie-OV)	OV10, OV14	http://identifiers.org/doi:10.17632/fstsb2xfsf.1
		OV25	http://identifiers.org/doi:10.17632/fstsb2xfsf.1; http://www.ebi.ac.uk/biostudies/studies/S-EPMC5725143?xr=true
		OV16, OV54	http://www.ebi.ac.uk/biostudies/studies/S-EPMC5725143?xr=true
Luxembourg Institute of Health - NORLUX laboratory	Glioma (LIH)	T16, T192, T233, T251, T341, T347, T470, T476, T591, T384	http://identifiers.org/ebi/bioproject:PRJNA627814
TRACE - Patient Derived Tumor Xenograft Platform, KU Leuven and UZ Leuven, Belgium	Breast Cancer, Cutaneous Melanoma, Uterine Cancer (TRACE)	MEL0029LY	https://www.ncbi.nlm.nih.gov/geo/query/acc.cgi?acc=GSE70180
		MEL0002LY	https://www.ncbi.nlm.nih.gov/geo/query/acc.cgi?acc=GSE116237
Vall d’Hebron Institute of Oncology, Spain	Colorectal Cancer (VHIO-CRC)	M001, M043	https://www.ncbi.nlm.nih.gov/geo/query/acc.cgi?acc=GSE96528
University Medical Center Groningen, Netherlands	Ovarian Cancer (UMCG)	UMCGOVPDX81, UMCGOVPDX68a, UMCGOVPDX37, UMCGOVPDX56, UMCGOVPDX84, UMCGOVPDX70, UMCGOVPDX79, UMCGOVPDX36	https://www.ebi.ac.uk/biostudies/studies/S-EPMC4594124?xr=true

Users can navigate to a model/patient/tumor page or to the data of interest by clicking on the unique PDX Model ID or data links in each row. At the lower-right corner of the page, users find options to download search results in a tabular format or to export them to a dedicated instance of cBioPortal for Cancer Genomics [6, 7] for deeper investigation of available molecular data, as described in the next section. A pre-compiled instance of the EurOPDX cBioPortal, including all main datasets currently stored in the Hub is publicly available at https://cbioportal.europdx.eu/.

A video tutorial containing the Portal functionalities can be found on EurOPDX RI YouTube channel next to other informative videos, please see https://youtu.be/l2AfjjcfT2Y.

### Tool chaining and consistent data availability

While the Data Portal allows to search and select models and samples according to certain criteria, the cBioPortal provides additional visualization and analysis capabilities. To link these two services, we leveraged the thorough design of the Data Hub API to extract a model selection in Data Portal and create a dedicated cBioPortal populated only with the data associated to the specific models selected. In order to speed up the startup time of such cBioPortal personalized to a user’s interest, snapshots of partially prepared (namely populated with all static data) cBioPortal Docker containers are reused.

## Conclusions

The EurOPDX Data Portal provides essential search functionality as well as complex molecular data analysis and visualization for cancer researchers interested in accessing PDX mice models. In particular, the Portal is the primary entry point for users interested in the free-of-charge research services offered by the EurOPDX RI. Indeed, from the perspective of the user looking for PDX models that are relevant to his/her research, retrieval of this information in the Portal serves as a background to request specific trans-national access services provided by the EurOPDX RI -- frozen PDX tumour samples shipment, in vivo studies on the selected PDX model(s), deposition of PDX model(s) (unlike the open access to data in the Portal, these services may be subject to further restrictions). The Portal is populated with curated data, special attention is paid to their thorough harmonization so that queries across multiple studies are possible. The procedure of data curation is well-defined and automated as much as possible to both speed up the process and avoid unintentional human errors. The data are shared among the Portal components, making sure by technical means that they are always mutually consistent.

At the time of writing this article, the Portal offers data on 1010 PDX models referring to 7 distinct cancer types, and the number models is expected to grow steadily. As such, the Portal provides the technical prerequisites to achieve the principal missions of the EurOPDX Consortium, which is to enable data sharing among the partners as well as to the broader research community, thereby improving the efficiency of PDX research and reducing duplication of efforts and unnecessary animal experiments.

## Data Availability

Most datasets included in the EurOPDX Data Portal are available after free authentication. The EurOPDX Data Portal is open however the downloading and re-use of data is subject to an Acceptable Use Policy and Conditions of Use (https://dataportal.europdx.eu/about/aup) as PDX models & associated data hosted by the EurOPDX Data Portal remain the property of the PDX developers, who retains all rights, title, and interest in and to the PDX models and data. Table 3 lists the original datasets that are publicly available, including their links. Besides those, several datasets included in Data Portal (IRCC-CRC, IRCC-GC, UOC-BC, Curie-BC, Curie-LC, NKI, VHIO-BC, UOM-BC) were not published yet. They are available on request from their providers, who can be contacted with the “Contact provider” button on each sample detail page in the portal.

## Abbreviations

API: Application Programming Interface
ChEBI: Chemical Entities of Biological Interest
ChEMBL: Chemical database of bioactive molecules with drug-like properties maintained by EMBL-EBI
CivicDB: Clinical interpretation of variants in cancer
COSMIC: Catalogue of Somatic Mutations in Cancer
DB: DataBase
EMBL-EBI: European Molecular Biology Laboratory - European Bioinformatics Institute
ETL: Extraction-Transformation-Loading
HGVS: Human Genome Variation Society
ID: Identifier
NCIt: National Cancer Institute thesaurus
OpenCravat: Open Custom Ranked Analysis of Variants Toolkit
PDX: Patient-derived Xenograft
PDX-MI: PDX-Minimal Information
REST: REpresentational State Transfer
RI: Research Infrastructure
VEP: Variant Effect Predictor

## Footnotes

1
https://dataportal.europdx.eu/

2
http://pdxfinder.org/

3
https://dataportal.europdx.eu/search

4
https://cbioportal.europdx.eu/

5
https://github.com/pdxfinder

6
https://www.apache.org/licenses/LICENSE-2.0

7
https://www.ensembl.org/info/docs/tools/vep/index.html

8
https://varnomen.hgvs.org

9
https://app.swaggerhub.com/apis-docs/RadimPesa/datahub/v1

10
http://www.genomecruzer.com/

11
https://dataportal.europdx.eu/search

12
https://edugain.org

13
https://www.europdx.eu/europdxri-ta

## References

[1] Invrea F, Rovito R, Torchiaro E, Petti C, Isella C, Medico E (2020). Patient-derived xenografts (PDXs) as model systems for human cancer. Curr Opin Biotechnol.

[2] Byrne AT, Alférez DG, Amant F, Annibali D, Arribas J, Biankin AV, Bruna A, Budinská E, Caldas C, Chang DK, Clarke RB, Clevers H, Coukos G, Dangles-Marie V, Eckhardt SG, Gonzalez-Suarez E, Hermans E, Hidalgo M, Jarzabek MA, de Jong S, Jonkers J, Kemper K, Lanfrancone L, Mælandsmo GM, Marangoni E, Marine JC, Medico E, Norum JH, Palmer HG, Peeper DS, Pelicci PG, Piris-Gimenez A, Roman-Roman S, Rueda OM, Seoane J, Serra V, Soucek L, Vanhecke D, Villanueva A, Vinolo E, Bertotti A, Trusolino L (2017). Interrogating open issues in cancer precision medicine with patient-derived xenografts. Nat Rev Cancer.

[3] Sartore-Bianchi A, Trusolino L, Martino C, Bencardino K, Lonardi S, Bergamo F, Zagonel V, Leone F, Depetris I, Martinelli E, Troiani T, Ciardiello F, Racca P, Bertotti A, Siravegna G, Torri V, Amatu A, Ghezzi S, Marrapese G, Palmeri L, Valtorta E, Cassingena A, Lauricella C, Vanzulli A, Regge D, Veronese S, Comoglio PM, Bardelli A, Marsoni S, Siena S (2016). Dual-targeted therapy with trastuzumab and lapatinib in treatment-refractory, KRAS codon 12/13 wild-type, HER2-positive metastatic colorectal cancer (HERACLES): a proof-of-concept, multicentre, open-label, phase 2 trial. Lancet Oncol.

[4] Meehan TF, Conte N, Goldstein T, Inghirami G, Murakami MA, Brabetz S, Gu Z, Wiser JA, Dunn P, Begley DA, Krupke DM, Bertotti A, Bruna A, Brush MH, Byrne AT, Caldas C, Christie AL, Clark DA, Dowst H, Dry JR, Doroshow JH, Duchamp O, Evrard YA, Ferretti S, Frese KK, Goodwin NC, Greenawalt D, Haendel MA, Hermans E, Houghton PJ, Jonkers J, Kemper K, Khor TO, Lewis MT, Lloyd KCK, Mason J, Medico E, Neuhauser SB, Olson JM, Peeper DS, Rueda OM, Seong JK, Trusolino L, Vinolo E, Wechsler-Reya RJ, Weinstock DM, Welm A, Weroha SJ, Amant F, Pfister SM, Kool M, Parkinson H, Butte AJ, Bult CJ (2017). PDX-MI: minimal information for patient-derived tumor xenograft models. Cancer Res.

[5] Conte N, Mason JC, Halmagyi C, Neuhauser S, Mosaku A, Yordanova G, Chatzipli A, Begley DA, Krupke DM, Parkinson H, Meehan TF, Bult CJ (2019). PDX Finder: a portal for patient-derived tumor xenograft model discovery. Nucleic Acids Res.

[6] Cerami E, Gao J, Dogrusoz U, Gross BE, Sumer SO, Aksoy BA, Jacobsen A, Byrne CJ, Heuer ML, Larsson E, Antipin Y, Reva B, Goldberg AP, Sander C, Schultz N (2012). The cBio cancer genomics portal: an open platform for exploring multidimensional cancer genomics data. Cancer Discov.

[7] Gao J, Aksoy BA, Dogrusoz U, Dresdner G, Gross B, Sumer SO, Sun Y, Jacobsen A, Sinha R, Larsson E, Cerami E, Sander C, Schultz N (2013). Integrative analysis of complex cancer genomics and clinical profiles using the cBioPortal. Sci Signal.

[8] de Coronado S, Wright LW, Fragoso G, Haber MW, Hahn-Dantona EA, Hartel FW, Quan SL, Safran T, Thomas N, Whiteman L (2009). The NCI thesaurus quality assurance life cycle. J Biomed Inform.

[9] Griffith M, Spies NC, Krysiak K, McMichael JF, Coffman AC, Danos AM, Ainscough BJ, Ramirez CA, Rieke DT, Kujan L, Barnell EK, Wagner AH, Skidmore ZL, Wollam A, Liu CJ, Jones MR, Bilski RL, Lesurf R, Feng YY, Shah NM, Bonakdar M, Trani L, Matlock M, Ramu A, Campbell KM, Spies GC, Graubert AP, Gangavarapu K, Eldred JM, Larson DE, Walker JR, Good BM, Wu C, Su AI, Dienstmann R, Margolin AA, Tamborero D, Lopez-Bigas N, Jones SJ, Bose R, Spencer DH, Wartman LD, Wilson RK, Mardis ER, Griffith OL (2017). CIViC is a community knowledgebase for expert crowdsourcing the clinical interpretation of variants in cancer. Nat Genet.

[10] Pagel KA, Kim R, Moad K, Busby B, Zheng L, Tokheim C, Ryan M, Karchin R (2020). Integrated informatics analysis of cancer-related variants. JCO Clin Cancer Inform.

[11] Forbes SA, Beare D, Gunasekaran P, Leung K, Bindal N, Boutselakis H, Ding M, Bamford S, Cole C, Ward S, Kok CY, Jia M, De T, Teague JW, Stratton MR, McDermott U, Campbell PJ (2015). COSMIC: exploring the world’s knowledge of somatic mutations in human cancer. Nucleic Acids Res.

[12] Yates B, Braschi B, Gray KA, Seal RL, Tweedie S, Bruford EA (2017). Genenames.org: the HGNC and VGNC resources in 2017. Nucleic Acids Res.

[13] Davisson MT (1997). Rules and guidelines for genetic nomenclature in mice: excerpted version. Committee on Standardized Genetic Nomenclature for Mice. Transgenic Res.

[14] Hastings J, Owen G, Dekker A, Ennis M, Kale N, Muthukrishnan V, Turner S, Swainston N, Mendes P, Steinbeck C (2016). ChEBI in 2016: improved services and an expanding collection of metabolites. Nucleic Acids Res.

[15] Bento AP, Gaulton A, Hersey A, Bellis LJ, Chambers J, Davies M, Krüger FA, Light Y, Mak L, McGlinchey S, Nowotka M, Papadatos G, Santos R, Overington JP (2014). The ChEMBL bioactivity database: an update. Nucleic Acids Res.

[16] Kim S, Thiessen PA, Bolton EE, Chen J, Fu G, Gindulyte A, Han L, He J, He S, Shoemaker BA, Wang J, Yu B, Zhang J, Bryant SH (2016). PubChem substance and compound databases. Nucleic Acids Res.

[17] Merkel D (2014). Docker: lightweight Linux containers for consistent development and deployment. Linux J.

[18] Umbraco content management system. https://umbraco.com/. Accessed 20 July 2021.

[19] Linden M, Procházka M, Lappalainen I, Bucik D, Vyskocil P, Kuba M, Silén S, Belmann P, Sczyrba A, Newhouse S, Matyska L, Nyrönen T (2018). Common ELIXIR service for researcher authentication and authorisation. F1000Res.

